# Factors contributing to the variability of a predictive score for cranial cruciate ligament deficiency in Labrador Retrievers

**DOI:** 10.1186/s12917-017-1154-9

**Published:** 2017-08-14

**Authors:** Devin P. Cunningham, Ayman A. Mostafa, Wanda J. Gordan-Evans, Randy J. Boudrieau, Dominique J. Griffon

**Affiliations:** 1 Western University of Health Sciences, Pomona, CA 91766 USA; 2 Department of Small Animal Surgery, Faculty of Veterinary Medicine, Cairo University, Cairo, Egypt; 3 Veterinary Medical Center, University of Minnesota, Saint-Paul, MN USA; 4 Cumming’s School of Veterinary Medicine at Tufts University, Grafton, North, MA 01536 USA; 5 University of Missouri, Columbia, MO USA

## Abstract

**Background:**

We recently reported that a conformation score derived from the tibial plateau angle (TPA) and the femoral anteversion angle (FAA), best discriminates limbs predisposed to, or affected by cranial cruciate ligament disease (CCLD), from those that are at low risk for CCLD. The specificity and sensitivity of this score were high enough to support further investigations toward its use for large-scale screening of dogs by veterinarians. The next step, which is the objective of the current study, is to determine inter-observer variability of that CCLD score in a large population of Labrador Retrievers. A total of 167 Labradors were enrolled in this cross-sectional study. Limbs of normal dogs over 6 years of age with no history of CCLD were considered at low risk for CCLD. Limbs of dogs with CCLD were considered at high risk for CCLD. Tibial plateau and femoral anteversion angles were measured independently by two investigators to calculate a CCLD score for each limb. Kappa statistics were used to determine the extent of agreement between investigators. Pearson’s correlation and intraclass coefficients were calculated to evaluate the correlation between investigators and the relative contribution of each measurement to the variability of the CCLD score.

**Results:**

The correlation between CCLD scores calculated by investigators was good (correlation coefficient = 0.68 *p* < 0.0001). However, interobserver agreement with regards to the predicted status of limbs was fair (kappa value = 0.28), with 37% of limbs being assigned divergent classifications. Variations in CCLD scores correlated best with those of TPA, which was the least consistent parameter between investigators. Absolute interobserver differences were two times greater for FAAs (4.19° ± 3.15) than TPAs (2.23° ± 1.91).

**Conclusions:**

The reproducibility of the CCLD score between investigators is fair, justifying caution when interpreting individual scores. Future studies should focus on improving the reproducibility of TPA and FAA measurements, as strategies to improve the agreement between CCLD scores.

## Background

The cranial cruciate ligament is an important stabilizer of the canine stifle, during the stance and swing phases of gait. This passive restraint works in conjunction with the menisci to prevent cranial subluxation of the tibia during weight bearing [1]. In large breed dogs, cranial cruciate ligament deficiency (CCLD) is the leading cause of pelvic limb lameness and degenerative joint disease [2]. The reported incidence of CCLD in large breed dogs ranges from 1.6 and 4.9% and has gradually increased over the last 40 years [2–5]. Labrador Retrievers are predisposed to CCLD, with a reported incidence of 3.8-5.8% [3, 5–7]. Cranial cruciate ligament (CCLD) commonly affects both stifles, with 50% of Labradors developing contralateral CCLD within 5.5 months [8, 9].

The exact pathogenesis of CCLD is unknown but most likely multifactorial in origin. Fatigue failure secondary to abnormal gait mechanics and repetitive micro-trauma has been proposed to result from conformational defects of the pelvic limb [10]. Among those, tibial plateau angle (TPA) seems to play a controversial role [1, 11]. In one study, dogs with CCLD had a steeper TPA than dogs without CCLD (23.8^o^ vs 18.1^o^) [12]. Another, however, found no difference in TPA’s between Labradors with and without CCLD, 23.5+/−3.1^o^ and 23.6+/−3.5^o^ respectively [13].

Recently, we found that a combination of two morphometric characteristics, TPA and the femoral anteversion angle (FAA), best discriminate limbs predisposed to, or affected by CCLD, from those that are a low risk [14]. An equation was developed based on these two parameters, to calculate a score (CCLD score) designed to predict the risk of CCLD [14]. The FAA used to calculate the CCLD score is obtained using a previously described bi-planar technique, including a true mediolateral projection of the femur and an extended ventrodorsal projection of the pelvis [15–21]. The CCLD score is derived from measurements generally accessible to veterinary practices equipped with standard radiography. This score is intended to serve as a foundation for large-scale screening of dogs for CCLD, which would require reproducibility between clinicians.

The aim of this study was to explore the variability of the CCLD score. The first goal was to determine the correlation of the CCLD scores among clinicians. We hypothesized that the CCLD score might differ between investigators but that the magnitude of this change would be such that there would be good agreement between investigators. The second objective was to determine the relative influence of each parameter used in the CCLD score on the variability of the score. Based on our clinical experience, we hypothesized that the FAA would vary the most between investigators and would have a significant impact on the variability of CCLD score.

## Methods

### Dogs

Informed consent was obtained from the owners of adult purebred Labrador Retrievers. The study protocol was reviewed by an IACUC at all participating institutions, except for one. The IACUCs considered that the study was conducted during routine clinical procedures; thus, approval from those IACUCs was not necessary provided the owners gave written informed consent. For the other institution, the study was reviewed and approved by a clinical studies group. The control group (normal dogs) included Labrador Retrievers that were at least 6 years old, had no history or clinical signs of stifle disease, and for which orthopedic and radiographic examinations of the stifles were normal [13]. Limbs in these dogs were considered at “low risk for CCLD” [13, 14, 22, 23]. Dogs with unilateral or bilateral CCLD (affected dogs) were included in the study if they had no history of trauma and were confirmed to have CCLD at the time of surgery. Dogs with CCLD could be enrolled in the study after surgical treatment of the CCLD if radiographs of their unaltered tibia were available [14, 22]. Limbs with CCLD were considered at high risk for CCLD. Contralateral limbs without evidence of CCLD were also considered at high risk for CCLD, due to the reported prevalence of contralateral CCLD in large breed dogs [8, 9]. Age, body condition scores and gender of each dog were recorded.

### Radiographic evaluations

Digital radiographs were obtained from each limb of every dog enrolled in the study. Radiographs included mediolateral projections of the tibia and femur and an extended ventrodorsal view of the pelvis; a reference calibration marker was placed at the level of the femur or tibia in all radiographs. The TPA was measured using landmarks previously described [24]. The tangential technique described by *Reif* et al. [25] was used to measure the TPA when severe degenerative joint disease compromised the identification of landmarks. Limbs were excluded from scoring if the stifle joint was not positioned appropriately: flexion angle not equal to 90^o^ or if the fabellae and femoral condyles were not superimposed on the mediolateral projections. Similarly, the pelvis was evaluated for symmetry and full extension of the femur (comparable femoral length on both mediolateral and ventrodorsal projections). Limbs were also excluded from scoring if the: fabellae and femoral condyles did not overlap with each other on the mediolateral projection of the femur; fabellae were not symmetrically superimposed over the femurs on the ventrodorsal projection; patella was not centered over the trochlear groove.

FAA was calculated using the bi-planar, right angle triangle technique [17] previously described by Bardet et al. [26] and Montavon et al. [19]. Briefly, the FAA was measured based on the distances between the axis of the femur and the center of the femoral head on orthogonal radiographic views of the femur [26].

### Conformational scores

Conformationa scores were only calculated on limbs with a complete set of adequately positioned radiographs. Scoring was based on the equation developed from our previous work [14]:

CCLD score = −33.49 + 0.37(FAA) + 0.82(TPA)

Limbs with a CCLD score greater than −1.5 were predicted as high risk for CCLD, whereas those with a score lower than −1.5 were predicted at low risk for CCLD [15]. Each set of radiographs was scored independently by two investigators (DG and AM), unaware of the status of the limb.

### Data analysis

Two-sample t-tests were used to compare the age, body condition scores and genders between diseased and normal dogs. Scatter plots and Pearson’s correlation coefficients were used to explore the correlation between CCLD scores. The kappa statistic [26–29] was used to evaluate the extent of agreement between each investigators’ predicted status (high versus low risk for CCLD) based on the CCLD score calculated for each limb. Possible values of kappa statistics ranged from −1 to 1, with 1 indicating perfect agreement and −1 indicating perfect disagreement, and 0 indicating completely random agreement. Kappa values 0-0.20 indicated slight agreement, 0.21-0.40 indicated fair agreement, 0.41-0.60 indicated moderate agreement, 0.61-0.80 indicated substantial agreement, and 0.81-1 indicated almost perfect agreement [30].

To evaluate factors influencing the variability of the conformation scores between investigators, descriptive statistics (mean, standard deviation and coefficient of variation) of the absolute differences in CCLD score, TPA and FAA between the investigators were calculated. Pearson’s correlation coefficients were also calculated. Intraclass correlation coefficients (ICC) [29, 31, 32] were used to assess the interobserver reliability (the consistency of measurements taken by the two investigators, i.e., the extent to which the investigators were interchangeable). The ICCs were calculated via two-way random effects models, assuming that investigators were considered as being a random selection, and dogs were considered as being a random selection from all possible dogs. ICCs range from 0 to 1. Higher ICC values indicate higher interobserver reliability. Interobserver reliability is poor for ICC values less than 0.40, fair for values between 0.40 and 0.59, good for values between 0.60-0.74 and excellent for values between 0.75-1 [32, 33].

The 95% confidence intervals (CI) of the ICCs were calculated [31]. *P*-values less than 0.05 were considered statistically significant. All analyses were conducted using SAS version 9.3 (SAS institute, Inc., Cary, NC)**.**

## Results

A total of 167 Labrador Retrievers were enrolled in the study: 72 dogs were recruited from 4 veterinary practices on the West coast; 38 dogs were enrolled by one specialty practice in the Midwest; and 57 cases were recruited from a veterinary teaching hospital/referral practice on the East coast. The demographics of 166 dogs were available for this study (Table 1). As expected based on inclusion criteria, normal dogs were older than dogs with CCLD. After exclusion of limbs due to improper radiographic positioning, CCLD scores were calculated in 222 limbs.

**Table 1:** Age, body condition score (BCS), and gender of normal dogs and dogs with CCLD (Mean and SD)

	Normal dogs	Number of normal dogs	Dogs with CCLD	Number of CCLD dogs	*p*-value
Age (months)	108.9 ± 32.6	97	84.2 ± 30.7	69	< 0.0001
BCS	5.5 ± 0.9	97	5.61 ± 1.0	57	0.48
Female		8		1	
Female Spayed		43		39	
Male		8		2	
Male Neutered		38		26	

**Fig. 1:**
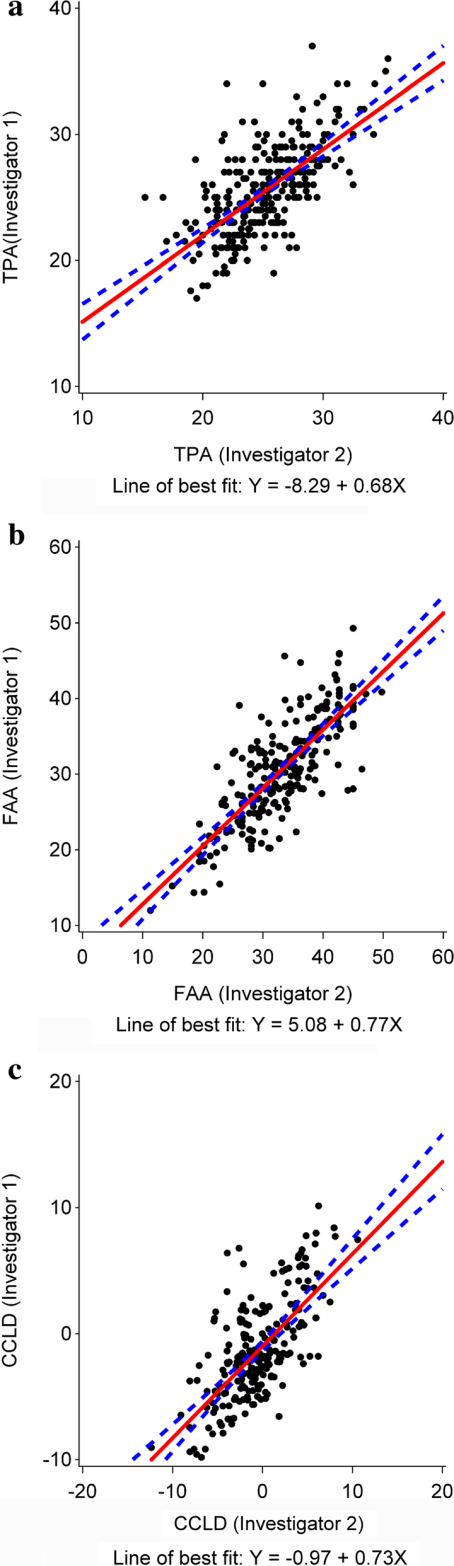
*Scatter plots* of TPAs (**a**), FAAs (**b**), and CCLD scores (**c**) measured by the 2 investigators. Note the difference in range of values between each plot

The correlation between CCLD scores measured by the two investigators was good, based upon Pearson’s correlation coefficient and ICC (Table 2), as well as the appearance of the scatter plot (Fig. 1). CCLD scores ranged from −9.8 to 10.2 (Fig. 1), averaging −1.35 (±3.96) and −0.52(±3.70) for investigators 1 and 2, respectively. Despite this good correlation between CCLD scores, the agreement between the resulting predicted status for each limb was fair, based on the kappa statistic (0.28 with a 95% CI of [0.16-0.40], *p* < 0.0001) (Table 3). Investigators agreed in predicting 67 limbs as high risk for CCLD, and 73 limbs as low risk for CCLD, while their scores diverged in 82 limbs. The observed proportion of agreement between the two investigators was 63.06% equally distributed between limbs predicted at high (30.2%) and low (32.9%) risk for the disease. The kappa statistic (0.28 with a 95% CI of [0.16-0.40], *p* < 0.0001) indicates a fair agreement in the predicted status for limbs between the two investigators.

**Table 2:** Variability of TPA, FAA, and CCLD scores, between the two investigators

	N	Differences between Investigator 1 and Investigator 2 (Mean ± SD)*	Coefficient of variation (%) of the difference between investigators	Pearson’s r coefficient of correlation (*p*-value)	Intraclass Correlation Coefficient (95% CI)
TPA (°)	289	2.23 ± 1.91	85.82	0.66 (< 0.0001)	0.66 (0.59, 0.72)
FAA (°)	231	4.19 ± 3.15	75.25	0.78 (< 0.0001)	0.78 (0.72, 0.83)
CCLD scores	222	2.58 ± 1.84	71.26	0.68 (< 0.0001)	0.68 (0.60, 0.74)

*Absolute values of differences. *N* = number of limbs

**Table 3:** Predicted status for limbs by two investigators

		Predicted status (Investigator 2)		Total
		High risk	Low risk	
Predicted status (Investigator 1)	High risk	67 (30.18)	22 (9.91)	89 (40.09)
	Low risk	60 (27.03)	73 (32.88)	133 (59.91)
	Total	127 (57.21)	95 (42.79)	222

Values represent the number of limbs predicted as a high or low risk for CCLD by each investigator. Numbers in parentheses are % of all limbs

A positive correlation was also found between both investigators’ measurements of TPA and FAA (*p* < 0.0001, Table 2). TPAs ranged from 15 to 37°, while FAAs ranged from 4 to 54° (Fig. 1). The magnitude of difference between investigators varied greatly between dogs (CV > 75%, Table 2), with an average of 2.23 ± 1.91° for TPA and 4.19 ± 3.15° for FAA (Table 2). The relative contribution of each radiographic measurement to the variability of CCLD scores in each group of limbs is represented in Table 4. Overall, there was a significant correlation (*p* < 0.0001) between the interobserver difference of CCLD score and differences in TPA (0.84), FAA (.70), mediolateral length (0.48) and ventrodorsal length (−0.42). Regardless of the status of the limb, the strongest correlation was between interobserver CCLD scores and their differences in TPA, followed by FAA. The absolute difference between TPAs measured by both investigators was slightly lower and the interobserver reliability for TPA was slightly better in normal than diseased limbs (Table 5). However, this difference was not statistically different as the 95% confidence intervals of ICCs overlapped between groups of limbs.

**Table 4:** Pearson’s correlation coefficients of the differences between investigators for CCLD score, and differences of TPA, FAA, mediolateral distance, and ventrodorsal distance

	Overall (*N* = 222)	Actual limb status		
		D (*N* = 46)	N (*N* = 151)	P (*N* = 25)
Difference of TPA	0.84 (< 0.0001)	0.78 (< 0.0001)	0.75 (< 0.0001)	0.87 (< 0.0001)
Difference of FAA	0.70 (< 0.0001)	0.67 (< 0.0001)	0.58 (< 0.0001)	0.81 (< 0.0001)
Difference of mediolateral distance	0.48 (< 0.0001)	0.21 (0.1601)	0.25 (< 0.0001)	0.75 (< 0.0001)
Difference of ventrodorsal distance	−0.42 (< 0.0001)	−0.49 (0.0005)	−0.45 (< 0.0001)	−0.27 (0.1902)

*Note: Numbers in parentheses are p-values, testing if the null hypothesis of zero correlation was true*

**Table 5:** Variability of TPA, stratified by normal and diseased limbs, between the two investigators

	N	Absolute value of difference between Investigator 1 and Investigator 2 (Mean ± SD)	Coefficient of variation (%) of the difference between investigators	Pearson’s r Coefficient of correlation (*p*-value)	Intraclass Correlation Coefficient (95% CI)
Normal	176	1.95 ± 1.44	73.69	0.69 (< 0.0001)	0.70 (0.61, 0.77)
Diseased	113	2.67 ± 2.42	90.88	0.68 (< 0.0001)	0.67 (0.55, 0.76)

*Absolute value

## Discussion

The CCLD score tested in our study was initially derived from data collected on 12 sound and 9 unilaterally CCL deficient Labrador Retrievers [14, 22, 34–37]. Similarly to our current study, hind limbs of normal dogs over six years of age were considered as non-predisposed to CCL deficiency and the contralateral limbs of CCL deficient dogs were classified as predisposed to CCLD. [14, 22, 34–37] A Receiver Operating Curve (ROC) analysis was used to assess the discriminating properties of conformation parameters for several combinations. A score (CCLD score) combining tibial plateau angle (TPA) and femoral anteversion angle (FAA) measured on radiographs was optimal for discriminating predisposed and non-predisposed limbs. This relatively small population was used to develop the CCLD score and therefore could not serve to validate the predictive value of this equation. We have consequently reported on the predictive value of this equation on the same 167 Labrador Retrievers included in the study described here [38]. In this population, we confirmed that TPA, FAA, and CCLD scores were greater in limbs at “high risk for CCLD” than in normal limbs. The sensitivity and specificity of the CCLD score reached 87% and 79%, while the negative and positive predictive values of the score were equal to 69% and 92%, respectively [38]. However, these findings were based on the measurements of a single investigator and did not address the variability of the CCLD score between investigators. Our current study, therefore, focuses specifically on the factors affecting the reproducibility of the scoring system, a prerequisite to its implementation in a large-scale screening program.

Although a good correlation was found between the CCLD scores measured by both investigators in this study, their level of agreement was fair (k = 0.28). In addition, the status of the limbs derived from their respective CCLD score diverged between observers in about 37% of cases. These findings prompt us to reject our first hypothesis. The level of interobserver agreement with regards to the CCLD score is at the low end of that reported in studies evaluating diagnostic tools clinically applied in small animal orthopedics. For example, radiographic screening for canine hip dysplasia based on hip extended views is routinely applied in dogs, with a reproducibility approximating 72% among experienced observers [34]. However, another study reported fair to moderate interobserver variability in detecting radiographic changes potentially associated with canine hip dysplasia, including osteosclerosis of the cranial acetabular edge, the presence of curvilinear caudolateral osteophytes, degenerative joint disease, circumferential femoral head osteophyte, and the diagnosis of suspected hip dysplasia [39]. In this study, the detection of osteosclerosis affecting the cranial acetabular edge was least reproducible (k = 0.23), while the identification of curvilinear caudolateral osteophyte and suspected hip dysplasia were the most reproducible diagnoses (k = 0.52). Overall, the authors concluded that the recognition of these specific signs was only fairly reliable and warranted caution when applied to official screening or surgical planning. The level of agreement of the CCLD score is also lower than that reported for the radiographic detection of elbow incongruity in dogs with elbow dysplasia (k = 0.45) [40]. This study explored the reproducibility of several subjective radiographic signs: the most reliable sign involved the detection of a radioulnar step (k = 0.72-0.8), while observers differed most when assessing the humeroulnar joint space on craniocaudal projections (k = 0.38) [40]. The subjective nature of signs used to test the reproducibility of detecting hip or elbow dysplasia may affect the detection of variations between investigators. Nonetheless, our findings are based on two investigators with extensive experience measuring CCLD scores. The inclusion of a greater number of less experienced clinicians, as expected in a large screening program, would further increase the variability of the CCLD score and derived limb status. We therefore recommend further investigations to improve the reproducibility of the CCLD score prior to its implementation in veterinary practices.

The factor whose variation correlated best with that of the CCLD score was the TPA (Pearson’s *r* = 0.84). Based on the Pearson’s r and intraclass correlation coefficients, this parameter was less reproducible between investigators than the FAA. This finding prompts us to suggest that TPAs impacted the variability of CCLD scores to a greater extent than FAAs, thereby contradicting our second hypothesis. The variability of TPA between investigators has been well established, with variation reaching 2 and 5°, within and between observers, respectively [41–43]. The absolute magnitude of variation of TPA between the 2 investigators in our study falls within this range, averaging 2.23° ± 1.91. Previous publications report that positioning of the limb and degree of degenerative joint disease (DJD) increase the variation of TPA, whereas no influence was detected when TPA was measured on digital images rather than traditional radiographs [25, 41, 42]. The positioning of limbs for our study was consistent with standard radiographic techniques previously recommended to measure FAA, as well as TPA in candidates for tibial plateau leveling osteotomy [22, 36, 43, 44]. The presence of DJD in the stifle complicates the identification of anatomic landmarks used to determine the TPA, especially the caudal extent of the tibial plateau [41, 43]. To palliate this limitation, we used a tangential method to measure the TPA in stifles with DJD [25]. This method requires the observer to estimate the position of the medial tibial plateau and draw a tangential line along this structure. The tangential method was found more variable than the conventional technique in healthy stifles but to the authors’ knowledge, has not been evaluated in diseased joints. In our study, the difference in TPAs between investigators was almost 1° greater in diseased than in normal dogs but was not statistically relevant. Further investigations are indicated to elucidate the respective influences of DJD and the radiographic method used to determine the TPA on the variability of CCLD scores.

As expected, FAA also influenced the variability of the CCLD score. Investigators were more consistent in their measurements of FAA than TPA and the lower coefficient assigned to FAA when calculating the CCLD score most likely mitigated the influence of FAA on the reproducibility of the CCLD score. The ICC and Pearson’s correlation coefficient evaluate consistency between investigators but do not imply absolute agreement. Indeed, the absolute difference between investigators averaged approximately 4°, almost twice the difference observed between investigators for TPAs. This finding may result from the larger range of FAAs compared to TPAs and explain our clinical impression regarding the variability of the measurement. Nonetheless, improving the reproducibility of FAA measurements appears to be another strategy to limit the variability of the CCLD score. The FAAs were measured with Reynold’s technique in our study, for consistency with the method used to develop the CCLD score [14]. An extended ventrodorsal projection of the pelvis and a true lateral projection of each examined femur are required to measure the distance between the femoral head and femoral axis in each plane. Of these two measurements, the distances measured on the ventrodorsal and mediolateral projections had a similar influence on the variability of the CCLD score. However, the extended ventrodorsal radiograph does not yield a true craniocaudal image of both femurs, as some inclination of each femur is expected. The resulting artifact consists of a variable degree of rotational mal-positioning, thereby influencing the calculations of FAA. A potential alternative would consist of replacing the ventrodorsal projection of the pelvis by a horizontal beam projection of the femur, previously proposed to obtain a true caudocranial view of the femur, with the dog in lateral recumbency [45].

## Conclusions

Despite a good correlation between CCLD scores, the resulting predicted risks of CCLD diverged between observers in 37% of limbs. This fair level of agreement warrants further investigations to improve the reproducibility of CCLD scores prior to large scale screening of dogs. TPA was less consistent than FAA between investigators and its variation had the majority of the impact on the CCLD score. Strategies limiting interobserver variability in TPAs are therefore likely to have the greatest impact on the reproducibility of CCLD scores. However, the range of FAAs and their absolute differences between investigators were larger than those of TPAs, justifying alternatives to also improve the reliability of FAAs measurements.

## Abbreviations

CCLD score: Conformational Score to predict CCLD
CCLD: Cranial Cruciate Ligament Deficiency
FAA: Femoral Anteversion Angle
PFA: Proximal Femoral Angle
TPA: Tibial Plateau Angle
